# Redefining the phenotype of ALSP and *AARS2* mutation–related leukodystrophy

**DOI:** 10.1212/NXG.0000000000000135

**Published:** 2017-02-15

**Authors:** Rahul Lakshmanan, Matthew E. Adams, David S. Lynch, Justin A. Kinsella, Rahul Phadke, Jonathan M. Schott, Elaine Murphy, Jonathan D. Rohrer, Jeremy Chataway, Henry Houlden, Nick C. Fox, Indran Davagnanam

**Affiliations:** From the Lysholm Department of Neuroradiology (R.L., M.E.A., I.D.), the National Hospital for Neurology and Neurosurgery; Department of Molecular Neuroscience (D.S.L., H.H.), UCL Institute of Neurology; the Leonard Wolfson Experimental Neurology Centre (D.S.L., J.A.K.), the National Hospital for Neurology and Neurosurgery, UCL Institute of Neurology; Dementia Research Centre (J.A.K., J.M.S., J.D.R., N.C.F.), Department of Neurodegeneration, UCL Institute of Neurology, UK; Department of Neurology (J.A.K.), St Vincent's University Hospital, University College Dublin, Ireland; Division of Neuropathology and Department of Neurodegenerative Disease (R.P.), Charles Dent Metabolic Unit (E.M.), Department of Neuroinflammation (J.C.), Neurogenetics Laboratory (H.H.), and Department of Brain Repair and Rehabilitation (I.D.), the National Hospital for Neurology and Neurosurgery, UCL Institute of Neurology, UK.

## Abstract

**Objective:**

To provide an overview of the phenotype of 2 clinically, radiologically, and pathologically similar leukodystrophies, adult-onset leukoencephalopathy with axonal spheroids and pigmented glia (ALSP) and alanyl-transfer RNA synthetase 2 mutation–related leukodystrophy (*AARS2*-L), and highlight key differentiating features.

**Methods:**

ALSP and *AARS2*-L cases were identified from the adult-onset leukodystrophy database at our institution. In addition, cases with imaging findings were identified from a literature review. The phenotypic features were determined by combining published cases with those from our database.

**Results:**

A combined total of 74 cases of ALSP and 10 cases of *AARS2*-L with neuroimaging data were identified. The mean age at onset was 42 years in ALSP and 26 years in *AARS2*-L. Cognitive and motor symptoms were the most common symptoms overall in both. Ovarian failure was exclusive to *AARS2*-L, present in all known female cases. Both ALSP and *AARS2*-L showed a confluent, asymmetric, predominantly frontoparietal, periventricular pattern of white matter disease with subcortical U-fiber sparing; pyramidal tract and corpus callosum involvement; and diffusion changes in the white matter which we have termed “deep white matter diffusion dots.” Central atrophy and corpus callosal thinning were prominent in ALSP and disproportionately mild in *AARS2*-L when present. ALSP also occasionally showed ventricular abnormalities and calcifications in the frontal periventricular white matter, features not seen in *AARS2*-L. *AARS2*-L demonstrates white matter rarefaction which suppresses on fluid-attenuated inversion recovery MRI sequences, a feature not seen in ALSP.

**Conclusions:**

ALSP and *AARS2*-L share similar clinical, imaging, and pathologic characteristics with key differentiating features that we have highlighted.

Adult-onset leukodystrophies are a rare (estimated prevalence of 2 in 100,000^1^) and diagnostically challenging group of conditions.^2^ MRI is pivotal in identifying the presence of a leukodystrophy; however, MRI findings are commonly etiologically nonspecific.^3^ Two leukodystrophies with similar clinical, imaging, and histopathologic^4^ phenotypes are adult-onset leukoencephalopathy with axonal spheroids and pigmented glia (ALSP), the most common adult-onset leukodystrophy,^5^ and a novel leukoencephalopathy due to autosomal recessive mutations in the mitochondrial alanyl–transfer RNA (tRNA) synthetase 2 gene (*AARS2*-L).^6^ ALSP was previously referred to as hereditary diffuse leukoencephalopathy with axonal spheroids (HDLS) or pigmentary orthochromatic leukodystrophy (POLD);^7,8^ however, ALSP is now preferred as the unifying term for leukodystrophies due to autosomal dominant mutations in the colony-stimulating factor receptor 1 (*CSF1R*) gene.^5^ Mutations in the *CSF1R* gene occur in the tyrosine kinase domain of the colony-stimulating factor receptor 1 which is primarily expressed in microglia in the CNS and results in microglial dysfunction in ALSP.^9,10^ Mutations in the *AARS2* gene cause errors in the mitochondrial aminoacyl tRNA synthase gene responsible for encoding alanine onto mitochondrial tRNA during mitochondrial translation^11^ and has been recently found to be the cause of an ovario-leukodystrophy^6^ and an infantile cardiomyopathy.^11^ The aim of this article is to summarize the characteristic imaging appearances of ALSP and *AARS2*-L and highlight their phenotypic similarities and discriminating clinical and imaging features.

## METHODS

### Standard protocol approvals, registrations, and patient consents.

This review was performed with the approval of the University College London Hospital Trust Institutional Review Board. All patients included in our local cohort had written consent to participate.

### Local patient cohort.

Eight cases of genetically confirmed ALSP and 4 *AARS2*-L cases with imaging were identified from an adult leukodystrophy database maintained by the Adult-onset Leukodystrophy Group (ALG) at the National Hospital for Neurology and Neurosurgery, Queen Square, London. Brain histopathology was available in 2 of our patients with ALSP and in 1 patient with *AARS2*-L. Histopathologic specimens were reviewed by an experienced consultant neuropathologist with a special interest in white matter pathologies.

### Consensus review of local imaging.

A review of the imaging findings in our 12 patients was performed by a neuroradiology fellow (R.L.) and 2 experienced neuroradiologists (M.E.A. and I.D.). Images were reviewed on a PACS workstation using Agfa Impax 6 (Agfa-Gevaert N.V., Mortsel, Belgium) and 4 diagnostic 3mp monochrome Barco (Kortrijk, Belgium) monitors. The neuroradiologists were blinded to patient demographics and clinical information and assessed the imaging findings for each case according to the following categories: white matter involvement (signal characteristics, lobar predominance, symmetry, extent, involvement of subcortical U fibers, and periventricular white matter); involvement of the corpus callosum, pyramidal tracts, brainstem, basal ganglia, and cerebellum; atrophy; ventricular abnormalities; diffusion-weighted imaging (DWI) abnormalities; gradient echo and/or susceptibility weighted imaging (SWI) abnormalities; progression over time; calcifications on CT, and vascular abnormalities. The findings of each case were tabulated after a consensus review.

### Literature review.

A review of the published literature was performed in the English language through a search of PubMed using each of the following search terms: “axonal spheroids,” “leu(c)kodystrophy and spheroids,” “HDLS,” “pigmented glia,” “leu(c)kodystrophy and pigmentary,” “pigmentary orthochromatic leu(c)kodystrophy or POLD,” “ALSP,” “*CSF1R*,” “alanyl-tRNA synthetase 2,” and “*AARS2*.” For both ALSP and AARS2-L, only genetically confirmed cases which had imaging data were included in the analysis.

### Imaging phenotype.

The imaging findings in the articles reviewed were summarized and combined with the cases from our cohort to define the imaging phenotype for both ALSP and *AARS2*-L.

## RESULTS

### Local patient cohort.

Of the 8 cases of ALSP, 6 (cases 1–6 in table 1) had been previously reported,^12,13^ with the 2 remaining ALSP cases having not been previously described. All 4 cases of *AARS2*-L have been published.^4^ The demographic, clinical, and genetic information for these 12 cases is summarized in table 1. All patients with ALSP and *AARS2*-L in our local cohort had MRI of the brain; CT head studies were available in 3 patients with ALSP (table 1, ALSP cases 2–4) and 2 patients with *AARS2*-L (table 1, *AARS2*-L cases 1 and 2).

**Table 1:**
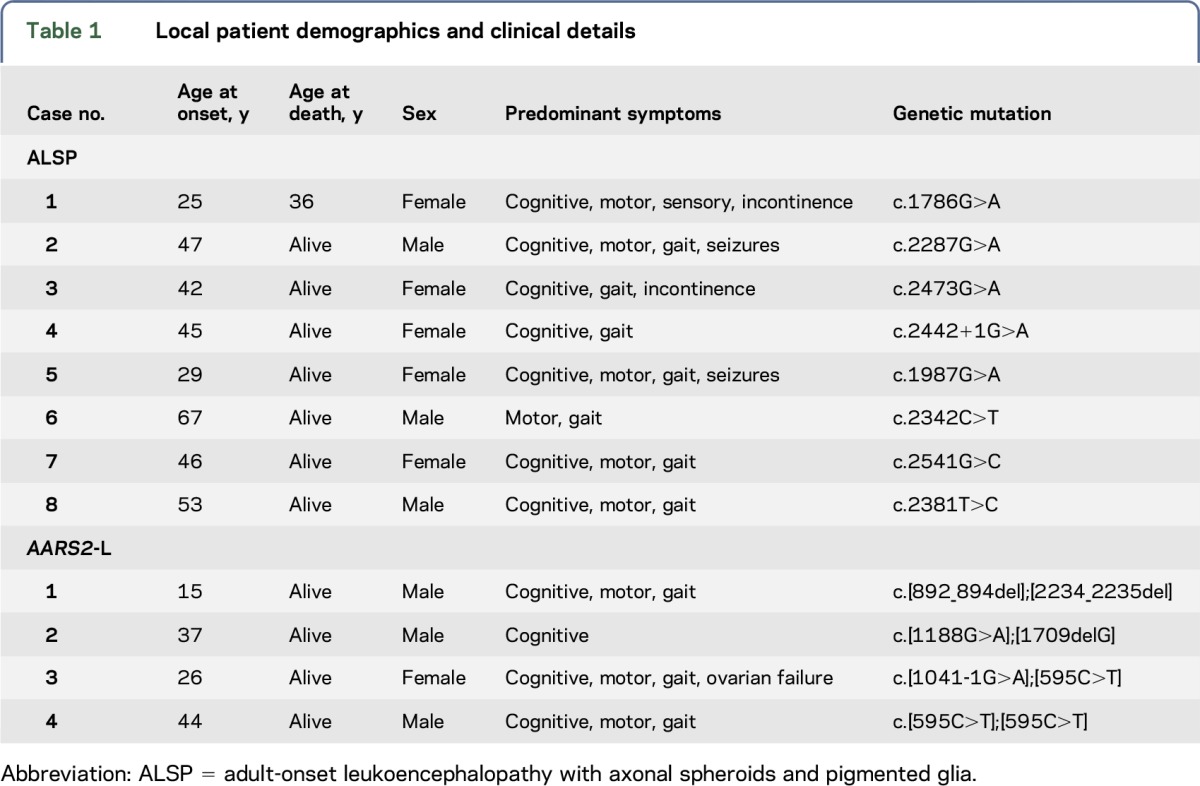
Local patient demographics and clinical details

### Histopathology.

Histopathology was available for ALSP cases 1 and 2 and for *AARS2*-L case 3 in table 1. Both ALSP cases 1 and 2 had histopathologic findings obtained from right frontal lobe biopsies which showed findings consistent with ALSP in case 1, table 1, and HDLS in case 2, table 1. In the original description of AARS2-L, pathologic findings from a muscle biopsy were reported without description of brain histopathology.^6^ A right parietal lobe biopsy in *AARS2*-L case 3 in table 1 showed histopathologic findings consistent with ALSP with numerous axonal spheroids and pigmented glia and has been described in greater detail in the original report of the case.^4^ *CSF1R* gene testing in this patient showed no pathogenic mutation, and mutations in the *AARS2* gene were instead identified.

### Literature review.

A total of 41 original articles were identified which contained imaging data, 40 articles for ALSP and 1 article for *AARS2*-L. This yielded a total of 103 cases of ALSP (including 2 previously undescribed cases from our institution) and 10 cases of *AARS2*-L. Twenty-nine cases of ALSP were excluded from the analysis because of the lack of a confirmed *CSF1R* mutation, this left at a total of 74 genetically confirmed ALSP cases. The summarized clinical, imaging, and pathologic data for all genetically confirmed ALSP and *AARS2*-L cases from our institution and the literature are presented in table 2.

**Table 2:**
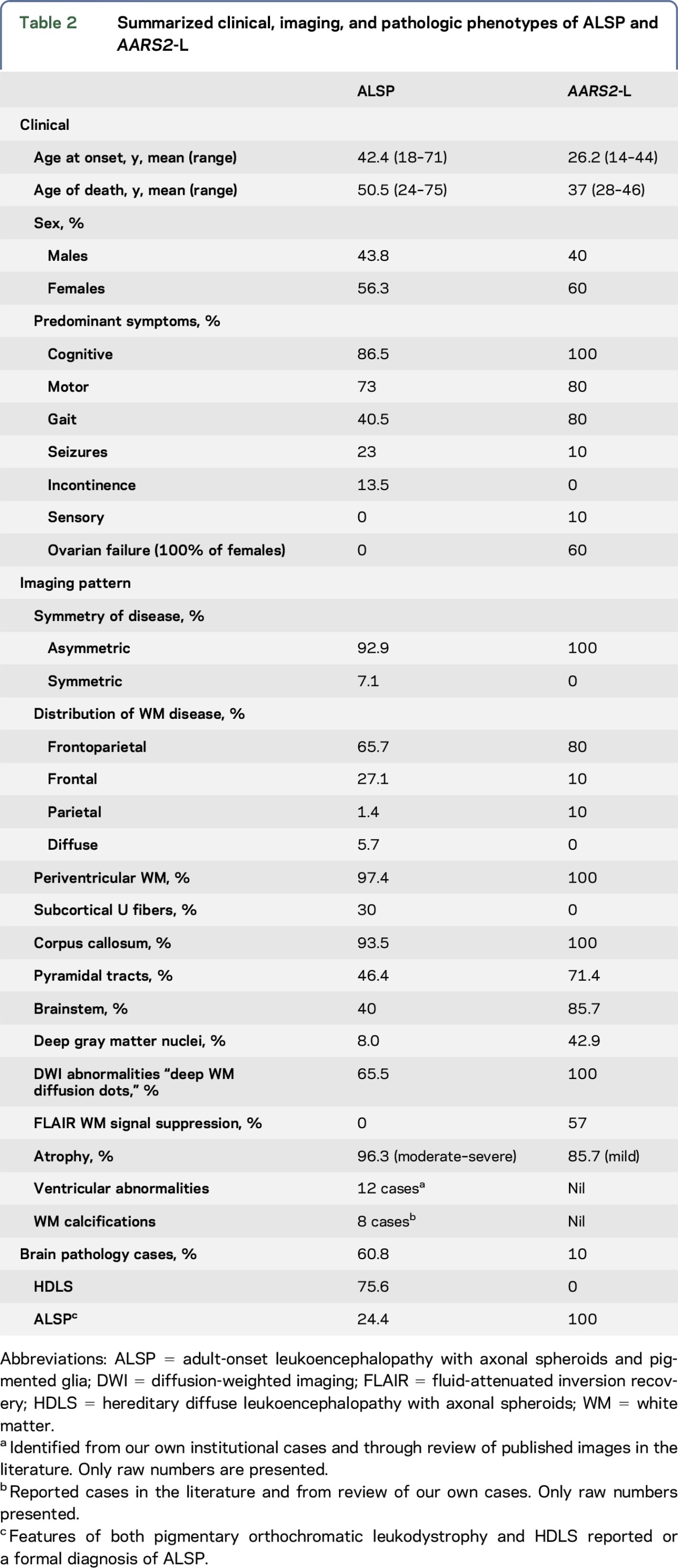
Summarized clinical, imaging, and pathologic phenotypes of ALSP and *AARS2*-L

### Imaging phenotype.

The imaging phenotypes were determined by combining the information available from imaging descriptions in the literature with the cases from our institution. The denominators below represent the total number of cases where information regarding each particular imaging descriptor was present.

### ALSP.

#### White matter signal.

**Figure 1:**
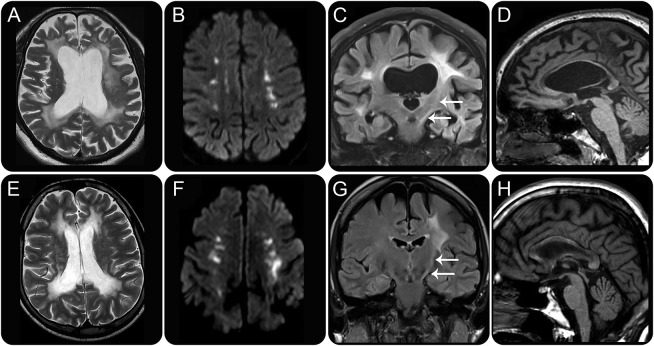
Imaging similarities between ALSP and *AARS2*-L Adult-onset leukoencephalopathy with axonal spheroids and pigmented glia (ALSP) MRI findings are shown in upper image panels A–D. *AARS2*-L MRI findings are shown in lower image panels E–H. (A and E) Axial T2-w MRI sequence in ALSP case 8, table 1, and *AARS2*-L case 1, table 1, respectively, showing similar confluent slightly asymmetric frontoparietal predominant white matter T2-w hyperintensity, with more atrophy in ALSP. (B and F) Axial diffusion-weighted imaging b1000 trace image in ALSP case 1, table 1, and *AARS2*-L case 4, table 1, respectively, illustrating near-identical linearly arranged punctate and partly confluent hyperintense “deep white matter diffusion dots”. (C and G) Coronal fluid-attenuated inversion recovery sequence in ALSP case 8, table 1, and *AARS2*-L case 3, table 1, respectively, both showing hyperintense involvement of the left corticospinal tract (white arrows). (D and H) Sagittal T1-w sequence in case 8, table 1, and *AARS2*-L case 1, table 1, illustrating focal low-signal corpus callosum involvement with more marked thinning of the corpus callosum in ALSP.

White matter hyperintensity was present on T2-weighted (T2-w) sequences in all patients with data (74/74). Of those with T2-w fluid-attenuated inversion recovery (FLAIR) imaging, none showed suppression of white matter signal to suggest white matter rarefaction. Of the 16 patients with T1-weighted (T1-w) imaging, all showed hypointensity in affected regions (figure 1, A, D, and E).

#### Distribution of white matter abnormality.

Predominance of the white matter signal abnormality was most commonly seen in a frontoparietal distribution (46/70), followed by a frontal predominant distribution (19/70). Diffuse involvement was seen in 4/70, with a parietal predominant distribution present in only 1/70. Of those with reports of symmetry or asymmetry, 39/42 had asymmetric white matter signal changes. A substantial majority of patients showed confluent white matter signal changes (30/33) as opposed to patchy involvement (3/33). Subcortical U fibers were involved in 9/30 patients, and it was noted in our patient cohort that the U fibers are generally spared until very late in the disease time course. Conversely, the periventricular white matter tends to be involved in most cases (37/38). However, in our patient cohort, it was noted that the immediate periependymal periventricular white matter tends to show a thin rim of sparing until later in the disease course.

#### Corpus callosum involvement.

Involvement of the corpus callosum was present in 58/62 cases, with generalized callosal thinning present in 36/58 (figure 1D). The splenium was most commonly involved (12/13), followed by the genu (8/13) and the callosal body (7/13), with the location of callosal involvement spatially linked to the location of deep white matter disease.

#### Pyramidal tract involvement.

Involvement of the pyramidal tracts was present in 13/28 (figure 1C). The pyramidal tract signal changes tended to occur later in the natural history of the disease in our patient group.

#### Deep gray matter nuclei and cerebellar involvement.

The deep gray matter nuclei are seldom involved in ALSP (2/25). These include a case which showed basal ganglia calcifications^14^ and another case which described striatal volume loss.^15^ Cerebellar involvement has only been described in a single case where the cerebellar peduncles were involved,^16^ as a rule, however, the cerebellum tends to be spared (22/23).

#### DWI abnormalities.

Abnormally high signal on the DWI trace images was described in 19/29 patients with diffusion data, suggesting that diffusion abnormalities are seen in a majority of patients. On apparent diffusion coefficient (ADC) maps, these areas either exhibit truly restricted diffusion or, as seen in some of our cases, diffusivity approximates that of normal white matter. The diffusion abnormalities are punctate and are most commonly seen in a linear arrangement, with lesion clusters aligned parallel to the ependymal surface of the lateral ventricles with a predisposition for the corona radiata (14/19) and centrum semiovale (10/19). DWI abnormalities may also arise in the splenium of the corpus callosum, although this is seen less commonly (4/19). In our patient cohort, we have observed that in contrast to diffusion abnormalities secondary to conventional infarction, these punctate areas of punctate DWI hyperintensity often persist over time, sometimes enlarging and coalescing. Other authors have described DWI abnormalities with a similar temporal evolution in ALSP^15,17,18^ which correlates with the temporal observations we have made in our patient group. To summarize the diffusion findings, we have coined the term “deep white matter diffusion dots,” and the findings are illustrated in figure 1B.

#### Atrophy.

Almost all patients (52/54) showed atrophy. In our cohort, the degree and location of atrophy correlated with the severity and location of white matter signal abnormality and was progressive over time (figure 1A).

#### Ventricular abnormalities.

**Figure 2:**
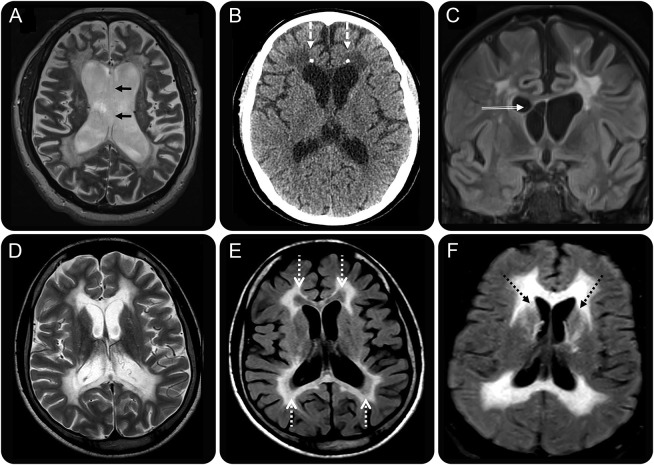
Imaging differences between ALSP and *AARS2*-L Adult-onset leukoencephalopathy with axonal spheroids and pigmented glia (ALSP) MRI findings are shown in upper image panels A–C. *AARS2*-L MRI findings are shown in lower image panels D–F. (A and D) Axial T2-w MRI sequence in ALSP case 8, table 1, and *AARS2*-L case 1, table 1, respectively, showing more severe atrophy in ALSP, despite a similar degree of white matter signal abnormality in both. Image panel (A) also shows a cavum septum pelludicum with a septal perforation (black arrows). (B) Axial unenhanced CT in ALSP case 4, table 1, illustrating frontal periventricular calcifications (white arrows). (C) Coronal fluid-attenuated inversion recovery (FLAIR) MRI image in ALSP case 7, table 1, showing linear septations in the frontal horn of the right lateral ventricle (compound white arrow). (E) Axial FLAIR MRI in *AARS2*-L case 1, table 1, showing suppression of white matter signal adjacent to the frontal horns and trigones of the lateral ventricles (dotted white arrows). (F) Axial FLAIR MRI in *AARS2*-L case 2, table 1, shows hyperintense signal in the heads of the caudate nuclei (black dotted arrows).

An observation in our cohort of patients was the presence of ventricular abnormalities. Ventricular septations were seen in 3/8 patients (figure 2C) and were commonly located within the frontal horns or near the foramen of Monro. Other ventricular abnormalities observed were septum pellucidum fenestrations (1/8, figure 2A) and a cavum septum pellucidum and/or cavum vergae (5/8 patients). This had been previously noted in 4 patients in a large CSF1R series,^16^ and upon review of the published images of ALSP, a cavum septum pellucidum or cavum vergae was evident in a further 6 patients.^19,20^ Ventricular enlargement was seen in almost all patients with data (26/27) and was proportional to the degree of volume loss.

#### Calcifications.

In total, calcifications have been reported in 8 patients^10,14,21,22^; these were most commonly reported to be located in the periventricular white matter adjacent to the frontal horns.^10,14,22^ Three of our cases had CT data and only one of those showed calcifications which were similarly present in the periventricular white matter adjacent to the frontal horns (figure 2B).

#### Vascular.

Five of 8 patients in our series had vascular imaging, including digital subtraction cerebral angiography in case 1, table 1. None of our patients showed vascular abnormalities, and no vascular abnormalities have been reported in the literature in association with ALSP. Of those patients in our group who had either T2* or SWI, none showed evidence of microhemorrhages.

#### Enhancement.

Of the 6 cases where there was enhanced imaging, no enhancement was observed.

### *AARS2*-L.

#### White matter signal.

All patients showed T2-w hyperintense, T1-w hypointense white matter signal abnormalities (figure 1, E, G, and H). Of those patients with FLAIR data, 4/7 showed suppression in the periventricular regions, presumably as a result of white matter rarefaction (figure 2E). These areas of FLAIR suppression did not show substantial volume loss, which is a feature similar to that seen in vanishing white matter disease.^23^

#### Distribution of white matter abnormality.

Eight of ten patients showed predominant involvement of the frontoparietal white matter. The remaining 2 patients showed frontal and parieto-occipital predominant involvement. Involvement was asymmetric in all patients with data (7/7) and was most commonly confluent (6/7) rather than patchy. Subcortical U fibers were always spared, and the periventricular white matter was always involved (figure 1E).

#### Corpus callosum involvement.

All patients with data showed involvement of the corpus callosum (7/7), figure 1H. The splenium was always involved with genu, and body involvement was present in 5/7 patients. The corpus callosum showed only mild thinning (6/7), much less severe than that seen in ALSP (figure 1H).

#### Pyramidal tract involvement.

Five of 7 patients showed pyramidal tract involvement; this was most commonly seen to involve the posterior limb of the internal capsule and the corticospinal tract in the brainstem (figure 1G). The involvement of the pyramidal tracts was asymmetric. One of 7 patients showed specific involvement of the frontopontine tract.^6^

#### Deep gray matter nuclei and cerebellar involvement.

Three of 7 patients showed T2-w hyperintensity in the caudate heads (figure 2F). One of 7 showed severe atrophy of the cerebellum.

#### Diffusion abnormalities.

**Figure 3:**
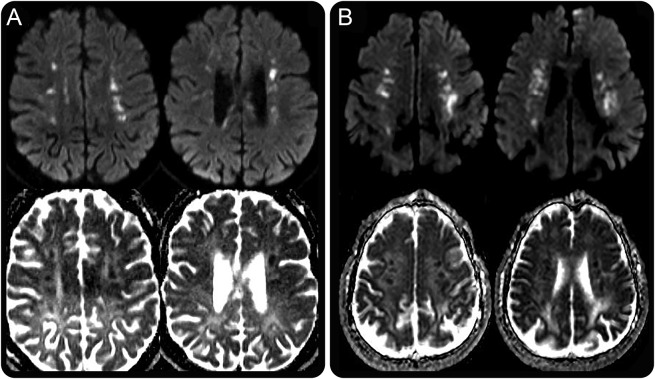
“Deep white matter diffusion dots” in ALSP and *AARS2*-L Axial diffusion-weighted imaging b1000 trace sequences (top row) and axial apparent diffusion coefficient sequences (bottom row) in adult-onset leukoencephalopathy with axonal spheroids and pigmented glia (ALSP) (A) and *AARS2*-L (B) showing linearly arranged, punctate, and partly confluent deep white matter areas of restricted diffusion arranged parallel to the ependymal surface.

All patients with diffusion data (5/5) showed restricted diffusion, with similar “deep white matter diffusion dots” as seen in ALSP, with linear, punctate, and partly confluent areas of restricted diffusion seen in the corona radiata and centrum semiovale with the lesion clusters aligned roughly parallel to the ependymal surface of the lateral ventricles (figure 3B). Restricted diffusion was seen in the corpus callosum of 3/5 patients, with the genu, body, and splenium all involved with equal frequency. Progression information was available in only one patient (case 3, table 1) in whom there was no temporal change in the diffusion during a 7-month follow-up period.

#### Atrophy.

Atrophy was present in 6 of 7 patients with data and was disproportionately mild compared with the degree of white matter signal abnormality. This is in contrast to ALSP in which atrophy was generally severe and proportional to the degree of white matter disease.

#### Ventricular abnormalities.

No ventricular abnormalities were evident in any of the patients.

#### Calcifications.

No calcifications were evident in the 2 patients who had CT data.

#### Vascular.

Case 3 in table 1, a 26-year-old female, showed a single microhemorrhage on a susceptibility weighted study. No gradient echo or SWI data were available for the other cases. No angiographic data were available in any of the 10 cases.

#### Enhancement.

No enhancement was detected in the 2 patients with enhanced MRIs.

## DISCUSSION

ALSP and *AARS2*-L are linked in many ways; they share common clinical features, imaging characteristics, and can share identical histopathologic appearances.^4^ There are, however, some important differentiating features, both on clinical and imaging grounds, which can help to favor one diagnosis over the other, many of which are presented in table 2. ALSP has shown to have an older age at onset, typically occurring in the fifth decade as opposed to *AARS2*-L where the onset of disease tends to occur in the third decade of life. All known females with *AARS2*-L present as an ovario-leukodystrophy with ovarian failure, a feature which is not seen in ALSP. Cognitive impairment and motor dysfunction are the most common presenting symptoms in both ALSP and *AARS2*-L, with both conditions also presenting with gait abnormalities, seizures, and incontinence. Sensory symptoms have been reported in 1 patient with *AARS2*-L and in no patients with ALSP.

There are many imaging features that ALSP and *AARS2*-L have in common. Both show frontoparietal predominant, slightly asymmetric, confluent T2-w hyperintense, T1-w hypointense white matter signal abnormalities which have a predilection for the periventricular and deep white matter and tend to spare the subcortical U fibers (figure 1, A and E). Both conditions show involvement of the corpus callosum; however, in ALSP, the callosal involvement is associated with severe thinning, which is not a feature of *AARS2*-L (figure 1, D and H). Both conditions demonstrate pyramidal tract involvement, typically in the posterior limb of the internal capsule and brainstem (figure 1, C and G).

The most striking shared imaging feature of ALSP and *AARS2*-L is the presence of DWI “deep white matter diffusion dots” which, to our knowledge, are not described in any other leukodystrophy. These DWI lesions are an imaging mimic of internal borderzone infarction and thus can potentially mislead clinicians into thinking that there is an underlying vascular pathology; however, the persistence and progression of these lesions over months to years differentiate them from areas of acute infarction. These areas of diffusion abnormality are multifocal, punctate, with the lesion clusters usually being roughly parallel to the ependymal surface of the lateral ventricles (figure 3, A and B). These lesions are typically located in the centrum semiovale and/or corona radiata but can also occur elsewhere especially along the corpus callosum or supratentorial pyramidal tracts. The diffusion abnormalities in ALSP have ADC values which appear similar to or slightly lower than normal white matter, whereas the foci observed in *AARS2*-L tend to exhibit more marked reductions in ADC, as would be seen in acute infarcts. Diffusion abnormalities are seen in a wide range of leukodystrophies, with restricted diffusion due to presumed intramyelinic edema observed in metachromatic leukodystrophy, globoid cell leukodystrophy, X-linked adrenoleukodystrophy, hyperhomocystinemias, Canavan disease, maple syrup urine disease, phenylketonuria, and leukodystrophy with brainstem and spinal cord involvement and high lactate.^24^ The patterns of diffusion abnormality in the aforementioned diseases are diverse; however, none demonstrate the characteristic pattern of “deep white matter diffusion dots,” which is seen in 66% of patients with ALSP and all known cases of *AARS2*-L with DWI included in this review.

An important differentiating feature on imaging is the suppression of rarefied periventricular white matter signal on T2-w FLAIR in *AARS2*-L, a feature which has not been described in ALSP (figure 2E). This appearance is similar to that seen in vanishing white matter disease, also an ovario-leukodystrophy with ovarian failure in female patients.^23^ The degree of atrophy is also a potential discriminating feature. In *AARS2*-L, atrophy is usually relatively mild compared with the degree of white matter involvement, whereas, in ALSP, the central atrophy is marked and is linked spatially to the areas of greatest MRI signal abnormality (figure 2, A and D). In some patients with *AARS2*-L, we have observed T2-w hyperintensity in the caudate heads, a feature absent in ALSP. An uncommon feature of ALSP which is not seen in *AARS2*-L is the presence of calcifications in the periventricular white matter adjacent to the frontal horns of the lateral ventricles, as was seen in one of our cases (figure 2B) and has been reported by others.^10,14,21^

Ventricular abnormalities, including ventricular septations, septum pellucidum fenestrations, or a cavum septum pellucidum and/or cavum vergae (figure 2, A and C), were evident in 7/8 ALSP cases in our institutional database and were not described in any cases of *AARS2*-L. Ventricular septations are usually seen in the setting of previous ventriculitis or intraventricular hemorrhage,^25^ and outside of this context is an unusual finding. Although conjecture, it is possible that the septations we have identified in patients with ALSP are secondary to an inflammatory response triggered within the ventricles. The presence of a cavum septum pellucidum or cavum vergae is of unknown significance, and when present in the general population is considered a normal variant. The frequent observation of a cavum septum pellucidum and/or cavum vergae in our case group may be incidental; however, the prevalence in the general population is felt to be in the vicinity of 12%–20%,^26^ which is in contrast to 63% in our local ALSP series. It is possible that damage to the white matter of the septum pellucidum allows communication of ventricular CSF with the potential space between the septal laminae, giving rise to a communicating form of a septum pellucidum, similar to that described in chronic traumatic encephalopathy.^27^

We have synthesized the phenotypic data for patients with ALSP and *AARS2*-L with the intention of highlighting both the similarities and differences between the clinical, imaging, and pathologic phenotypes of the 2 conditions. Distinguishing between ALSP and *AARS2*-L radiologically is of particular importance, given that the histopathologic findings are near identical in both^4^ and a brain biopsy in the absence of the correct genetic test may be misleading. Also recently, there has been a report of possible halted progression of ALSP in a *CSF1R* mutation–positive patient who underwent stem cell transplantation,^28^ potentially indicating a future therapeutic implication for correctly diagnosing these entities, although this is yet to be confirmed. As the genotypic spectrum of ALSP and *AARS2*-L expands, detailed genotype-phenotype correlations for each condition will be needed to determine the phenotypic significance of each mutation.

## GLOSSARY

ADC: apparent diffusion coefficient
ALG: Adult-onset Leukodystrophy Group
ALSP: adult-onset leukoencephalopathy with axonal spheroids and pigmented glia
CSF1R: colony-stimulating factor receptor 1
DWI: diffusion-weighted imaging
FLAIR: fluid-attenuated inversion recovery
HDLS: hereditary diffuse leukoencephalopathy with axonal spheroids
POLD: pigmentary orthochromatic leukodystrophy
SWI: susceptibility weighted imaging
tRNA: transfer RNA
